# Individualized prediction of post-acute pancreatitis diabetes mellitus by combining lipid metabolism and anatomical features

**DOI:** 10.1186/s13244-025-02039-w

**Published:** 2025-07-31

**Authors:** Ling Ling Tang, Qi Zhang, Shuang Yi Song, Nian Liu, Qing Lin Du, Shu Ting Zhong, Xiao Hua Huang

**Affiliations:** 1 Department of Radiology, Affiliated Hospital of North Sichuan Medical College, Nanchong, China; 2 School of Medical Imaging, North Sichuan Medical College, Nanchong, China

**Keywords:** Acute pancreatitis, Diabetes, Lipid metabolism, Pancreaticobiliary junction, Magnetic resonance cholangiopancreatography

## Abstract

**Objectives:**

To investigate the lipid metabolism and anatomical risk factors of post-acute pancreatitis diabetes mellitus (PPDM) and their value in individualized prediction.

**Materials and methods:**

A continuous retrospective analysis was conducted on 241 patients with acute pancreatitis (AP) treated in our hospital from January 2017 to December 2021. The type and angle of the pancreaticobiliary junction were measured on magnetic resonance cholangiopancreatography (MRCP) images, and baseline lipid metabolism indicators were collected. We evaluated the risk factors of PPDM using univariate and multivariate Cox proportional hazard analysis, established quantitative prediction models for PPDM, and evaluated the predictive value of lipid metabolism and features of the pancreaticobiliary junction.

**Results:**

Overall, 85 of 241 eligible patients (35.27%) ultimately developed PPDM. Univariate and multivariate analyses showed B-P type in pancreaticobiliary junction (*p* = 0.017), the angle of junction (*p* = 0.041), non-high-density lipoprotein (*p* = 0.029), alcohol index (*p* < 0.001), body mass index (*p* = 0.042), inflammatory frequency (*p* = 0.016), fasting blood glucose (*p* = 0.002), concomitant hypertension (*p* < 0.001) were important predictive factors for the occurrence of PPDM. The model that integrated imaging features of the pancreaticobiliary junction has a higher predictive performance than models without imaging features, with an AUC of 0.882 (95% CI, 0.836–0.930). The AUC of the combined model was 0.886 (95% CI, 0.841–0.932), and there was no statistical difference in AUC between the combined model and the pancreaticobiliary junction model (*p* = 0.340).

**Conclusion:**

The lipid metabolism and morphological characteristics of the pancreaticobiliary junction are additional risk factors for PPDM, and the quantitative prediction model shows moderate predictive performance.

**Critical relevance statement:**

The type and angle of the pancreaticobiliary junction based on MRCP are independent predictors of PPDM, which can quantitatively predict risk in the early stage.

**Key Points:**

- PPDM has an increasing incidence and poor prognosis, which requires early monitoring.
- Larger angles and B-P type in the pancreaticobiliary junction are risk factors for PPDM.
- Quantitative prediction of PPDM risk allows for early personalized prevention and treatment.

**Graphical Abstract:**

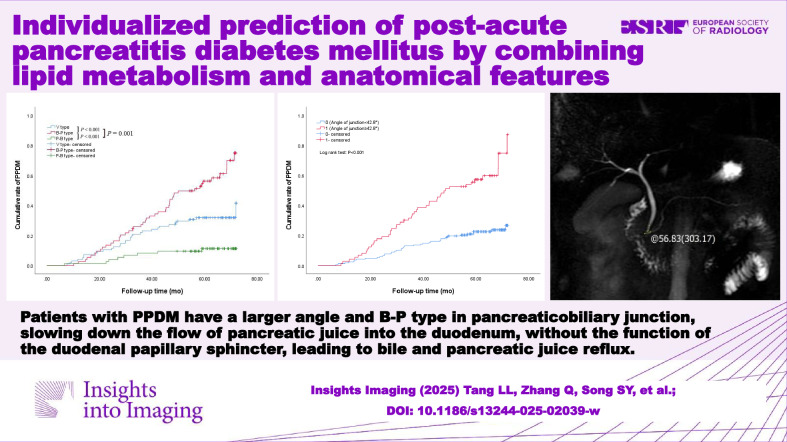

## Introduction

Acute pancreatitis (AP), one of the most common acute abdominal conditions clinically, is an inflammatory disease characterized by edema and necrosis of glandular tissue [1], which can easily lead to endocrine disorders. In recent years, the incidence of post-acute pancreatitis diabetes mellitus (PPDM) has gradually increased, which is characterized by poor blood glucose control, with an increased risk of pancreatic ductal adenocarcinoma in the later stage, bringing a heavy burden to patients and society [2–4]. Therefore, early identification of risk factors for PPDM provides a reference for clinical physicians to develop strategies to prevent or delay the occurrence and development of PPDM, thereby improving clinical prognosis.

With the improvement of the living standard and the change in diet structure, the role of lipid metabolism disorders in the pathogenesis and prognosis of AP is receiving increasing attention [5], with triglycerides (TG) being the focus of scholars’ exploration. Previous studies [6–8] found that patients with metabolic syndrome combined with obesity, hypertension, hyperglycemia, and hyperlipidemia have a higher risk of developing PPDM in the later stage. Pendharkar et al [9] also found that fat breakdown is an important pathogenesis of PPDM. However, abnormalities in other blood lipid indicators [10–12] may also affect the occurrence and development of PPDM, which has been overlooked in previous studies.

The pathogenesis of AP involves the destruction of the pancreatic self-defense system and the activation of autodigestion by enzymes. The defense mechanism of the pancreas is based on its anatomy and morphological structure. Our team’s preliminary research [13, 14] has found that the type and angle of the pancreaticobiliary junction are closely related to the occurrence and recurrence of AP. Therefore, we speculate that the morphological characteristics of the pancreaticobiliary junction can also serve as a risk marker for early prediction of PPDM.

To solve these problems, our study was to explore the influence of the lipid metabolism and the morphological characteristics of the pancreaticobiliary junction on PPDM, and then create a quantitative model to provide a scientific reference for clinical intervention measures.

## Materials and methods

### Patients

This retrospective study received institutional review board approval before the research was started. We conducted a continuous retrospective analysis of 638 AP patients diagnosed and treated in our hospital from January 2017 to December 2021, and follow-up occurred through telephone or medical documents until December 2023 to record the occurrence of diabetes. In line with the inclusion and exclusion criteria, a total of 241 patients were recruited.

The inclusion criteria were as follows: (1) all patients met the diagnostic standard of AP and underwent routine MRI and magnetic resonance cholangiopancreatography (MRCP) examinations 3 to 7 days after the onset of symptom, (2) patients in PPDM group met the diagnostic criteria of diabetes, (3) the interval between the new diagnosis of diabetes after the first AP is more than 3 months. The exclusion criteria were as follows: (1) previous history of prediabetes or diabetes (98 cases, 15.36%); (2) fasting or random blood glucose did not reduce to normal levels during hospitalization (77 cases, 12.07%); (3) history of chronic pancreatitis, pancreatic tumor, bile duct tumor, ampullary tumor or severe chronic wasting disease (102 cases, 15.99%); (4) poor MR image quality, incomplete clinical medical records, or loss of follow-up (112 cases, 17.55%); and (5) less than 18 years old (8 cases, 1.25%).

### Definition of AP and PPDM

The diagnosis of AP shall at least meet 2 of the following 3 items [15]: (1) sudden, severe, and persistent abdominal pain, which may be accompanied by back radiation pain; (2) serum amylase and/or lipase shall be at least 3 times higher than the upper limit of normal value; and (3) characteristic abdominal imaging findings consistent with AP. The patients were graded as mild, moderately severe, and severe based on the Atlanta 2012 classification.

PPDM was defined as having no previous diabetes base and being diagnosed as diabetes at least 3 months after the first AP, that is, meeting one of the following four items [16]: (1) typical diabetes symptoms and random blood glucose was greater than or equal to 11.1 mmol/L (≥ 200 mg/dL); (2) fasting blood glucose (FBG) was greater than or equal to 7.0 mmol/L (≥ 126 mg/dL); (3) 2-hour oral glucose tolerance test was greater than or equal to 11.1 mmol/L (≥ 200 mg/dL); and (4) glycated hemoglobin was greater than or equal to 6.5%.

### Data collection

We viewed the medical records of each enrolled patient from a hospital-based electronic database. The following baseline data were collected: (1) general characteristics, including gender, age, smoking index, alcohol index (AI), body mass index (BMI), and comorbidity (hypertension, fatty liver); (2) imaging features, including necrosis, the main location of the lesion, biliary stones, the type and angle of the pancreaticobiliary junction, local complications, and types of local complications; (3) laboratory data, including lipid metabolism indicators, blood calcium, FBG, white blood cells, percentage of neutrophils, C-reactive protein (CRP), serum pancreatic amylase, serum amylase, serum lipase; and (4) other indicators, including inflammation frequency (IF) and severity of AP.

### Measurement of indicators

Detailed information about MR scan equipment and scan parameters is presented in Supplementary S1. A radiologist with 8 years of experience in abdominal MRI diagnosis used the angle measurement tool in picture archiving and communication system (PACS) to measure the angle and evaluate the type of MRCP images, and another radiologist with 15 years of experience in abdominal MRI diagnosis conducted a review. In the MRCP image, we selected the junction of the pancreaticobiliary as the region of interest (ROI), measured the convergence angle of the main pancreatic duct and common bile duct with the junction of the pancreaticobiliary as the angle vertex, and the junction near the vertex turning point as both sides. The results of the angle were displayed on the MRCP image, and the type was recorded. The angle was measured three times separately, and the average value was calculated.

According to how the common bile duct and the main pancreatic duct converge into the duodenum, the confluence of the pancreaticobiliary can be divided into three types [13]: (1) V type, refers to the common bile duct and main pancreatic duct converging into the duodenal wall without a common pathway; (2) B-P type, which refers to the common bile duct draining into the main pancreatic duct and forming a common channel; (3) P-B type refers to the main pancreatic duct draining into the common bile duct and forming a common channel.

The venous blood samples before treatment were collected for examination within 12 h after admission. The blood lipids measurements were performed via a homogeneous enzymatic assay method, including serum TG, total cholesterol (TC), very low-density lipoprotein (VLDL), low density lipoprotein (LDL), high density lipoprotein (HDL), non high-density lipoprotein (NHDL), lipoprotein a, apolipoprotein A1 and apolipoprotein B100. NHDL [17] refers to the sum of cholesterol contained in other lipoproteins except HDL, which means that, in addition to LDL, it also includes VLDL and cholesterol contained in residual lipoproteins.

### Statistics

Statistical analyses were conducted by using SPSS 27.0 and X-tile 3.6.1. Continuous variables were presented as mean (standard deviation [SD]) or median and interquartile range (IQR) based on their distributions, and categorical variables were presented as numbers and percentages. The Kolmogorov-Smirnov method was applied to test the normality of the data. Independent sample *t*-tests and Mann–Whitney *U*-tests were used for continuous variables, when appropriate. The chi-square test was used for categorical variables. Obtaining the best cut-off value through the X-tile software. Univariate and multivariate Cox proportional hazard analysis were used to evaluate the risk factors for PPDM and the hazard ratio (HR) of risk factors. The Kaplan–Meier method was used to calculate the cumulative rate of PPDM. Using R (version 4.0.3, https://www.r-project.org/) to establish quantitative prediction models and visualize the combined model by nomogram, internal validation of the model was conducted using bootstrap equal to 1000 repeated samples with placement. The predictive performance of the model was evaluated using the receiver operator characteristic curve (ROC) and the area under the curve (AUC), and the consistency of the nomogram was evaluated using the calibration curve. The AUCs of the models were compared by the DeLong test. The difference was considered statistically significant at *p* < 0.05.

## Results

### Patient characteristics

The characteristics of 241 eligible patients with AP are listed in Tables 1–3. Among these cases, 85 (35.27%) patients eventually developed PPDM, with an interval of 6.80–71.82 [32.63 (IQR, 22.42–46.68)] months between AP and PPDM, while the remaining 156 (64.73%) in the nPPDM group, with a follow-up period of 32.67–72.00 [65.00 (IQR, 58.90–70.05)] months. The Spearman correlation analysis revealed a correlation coefficient of 0.069 between the angle and type of pancreaticobiliary junction, with no statistically significant difference (*p* = 0.289). Further subgroup analysis indicated that the correlation coefficient between the angle and type of junction was 0.109 in the nPPDM group (*p* = 0.177), while it was 0.293 in the PPDM group (*p* = 0.006). The BMI in the PPDM group (26.11 ± 3.63) was significantly higher than that in the nPPDM group (24.03 ± 3.56; *p* < 0.001). The median values of AI in the PPDM group and nPPDM group were 100 (IQR, 0–2500) and 0 (IQR, 0–87.5), respectively (*p* = 0.002). The proportions of patients with hypertension in the PPDM group and nPPDM group were 23.5% and 7.1%, respectively (*p* < 0.001). The median value of IF in the PPDM group was 2 times (IQR, 1–3 times), and that in the nPPDM group was 1 time (IQR, 1–2 times) (*p* < 0.001). The proportion of hemorrhagic necrosis in the PPDM group (22.35%) was higher than that in the nPPDM group (10.26%) (*p* = 0.011). In terms of the junction type, we found that the proportion of B-P type in the PPDM group (54.1%) was higher than that in the nPPDM group (17.3%) (*p* < 0.001). The median junction angle of the PPDM group and nPPDM group were 43.65° (IQR, 33.68°–53.05°) and 32.40° (IQR, 24.74°–40.27°), respectively (*p* < 0.001), and further analysis revealed that the angle of B-P type patients is greater than that of V type and P-B type patients. The proportions of local complications in the PPDM group (50.6%) were higher than those in the nPPDM group (34.0%) (*p* = 0.012), which was consistent with the difference in severe acute pancreatitis (SAP) between the two groups (*p* = 0.025).

**Table 1:** General characteristics of patients

	nPPDM group (*n* = 156)	PPDM group (*n* = 85)	*t*/χ^2^/*Z*	*p* value
Follow-up period	65.00 (58.90–70.05)	32.63 (22.42–46.68)		
Sex (male/female)	101/55	56/29	0.031	0.859
Age (years)	45 (37–55)	47 (38–57)	−1.094	0.274
Smoking index	0 (0–100)	0 (0–240)	−1.808	0.071
AI	0 (0–87.5)	100 (0–2500)	−3.037	**0.002**
BMI (kg/m^2^)	24.03 ± 3.56	26.11 ± 3.63	−4.308	**< 0.001**
Hypertension (*n*/%)	11/7.10%	20/23.50%	13.329	**< 0.001**
Fatty liver (*n*/%)	61/39.10%	39/45.90%	1.042	0.307
IF (times)	1 (1–2)	2 (1–3)	−5.337	**< 0.001**

Bold values are statistically significant

*BMI* body mass index, *IF* inflammation frequency, *AI* alcohol index

**Table 2:** Imaging characteristics of patients

	nPPDM group (*n* = 156)	PPDM group (*n* = 85)	χ^2^/*Z*	*p* value
Necrosis (*n*/%)	16/10.26%	19/22.35%	6.486	**0.011**
Main location of the lesion			10.412	**0.015**
Head	52/33.3%	22/25.9%		
Neck	39/25.0%	13/15.3%		
Body	50/32.1%	45/52.9%		
Tail	15/9.6%	5/5.9%		
Severity			7.365	**0.025**
MAP	60/38.5%	25/29.4%		
MSAP	78/50.0%	39/45.9%		
SAP	18/11.5%	21/24.7%		
Biliary stones (*n*/%)	44/28.20%	28/32.90%	0.589	0.443
Type of junction			43.800	**< 0.001**
V type	64/41.0%	31/36.5%		
B-P type	27/17.3%	46/54.1%		
P-B type	65/41.7%	8/9.4%		
Angle of junction (°)	32.40 (24.74–40.27)	43.65 (33.68–53.05)	−5.966	**< 0.001**
Local complications (*n*/%)	53/34.0%	43/50.6%	6.337	**0.012**
Type of local complications			10.550	**0.019**
APFC	48/30.8%	33/38.8%		
ANC	2/1.3%	2/2.4%		
PPC	2/1.3%	5/5.9%		
WON	1/0.6%	3/3.5%		
IPN	0/0.0%	0/0.0%		

Bold values are statistically significant

*MAP* mild acute pancreatitis, *MSAP* moderate severe acute pancreatitis, *SAP* severe acute pancreatitis, *APFC* acute peripancreatic fluid collection, *ANC* acute necrotic collection, *PPC* pancreatic pseudocyst, *WON* walled-off necrosis, *IPN* infected pancreatic necrosis

**Table 3:** Laboratory indicators of patients

	nPPDM group (*n* = 156)	PPDM group (*n* = 85)	*t*/*Z*	*p* value
TG (mmol/L)	1.80 (1.00–6.84)	4.90 (1.66–13.25)	−3.776	**< 0.001**
TC (mmol/L)	4.63 (3.90–5.91)	4.90 (4.01–6.78)	−1.398	0.162
VLDL (mmol/L)	0.97 (0.56–1.75)	1.25 (0.72–3.90)	−2.957	**0.003**
LDL (mmol/L)	2.42 (1.72–3.26)	2.21 (1.24–3.41)	−0.987	0.324
HDL (mmol/L)	1.02 (0.74–1.31)	0.87 (0.69–1.13)	−2.053	**0.040**
NHDL (mmol/L)	3.56 (2.76–4.70)	4.08 (3.09–5.74)	−2.643	**0.008**
Lipoprotein a (mg/L)	56.41 (25.90–201.70)	59.70 (16.40–159.90)	−0.827	0.408
Apolipoprotein A1 (g/L)	1.05 ± 0.27	0.98 ± 0.30	1.795	0.074
Apolipoprotein B (g/L)	0.72 ± 0.29	0.73 ± 0.32	−0.052	0.959
Blood calcium	2.31 (2.19–2.41)	2.28 (2.16–2.38)	−1.176	0.240
FBG (mmol/L)	6.90 (5.61–8.02)	7.92 (6.73–9.87)	−4.182	**< 0.001**
WBC (×10^9^/L)	12.28 (9.89–15.70)	13.32 (9.87–16.04)	−0.609	0.542
NEUT (%)	84.75 (79.75–89.00)	85.50 (80.70–89.65)	−0.684	0.494
CRP (mg/L)	28.71 (8.17–71.12)	37.81 (11.64–104.61)	−2.088	0.037
Amylopsin (U/L)	174.00 (65.10–665.15)	121.00 (59.05–399.00)	−1.321	0.187
Amylase (U/L)	212.00 (92.75–712.25)	151.70 (82.50–491.00)	−1.212	0.226
Lipase (U/L)	304.50 (95.00–1009.90)	229.60 (100.55–914.30)	−0.416	0.678

Bold values are statistically significant

*TG* triglycerides, *TC* total cholesterol, *VLDL* very low-density lipoprotein, *LDL* low density lipoprotein, *HDL* high density lipoprotein, *NHDL* non high-density lipoprotein, *FBG* fasting blood glucose, *WBC* white blood cell, *NEUT* percentage of neutrophils, *CRP* C-reactive protein

For laboratory data, the median TG of the PPDM group (4.90 mmol/L; IQR, 1.66–13.25 mmol/L) was significantly higher than that nPPDM group (1.80 mmol/L; IQR, 1.00–6.84 mmol/L; *p* < 0.001). The median VLDL of the PPDM group (1.25 mmol/L; IQR, 0.72–3.90 mmol/L) was significantly higher than that nPPDM group (0.97 mmol/L; IQR, 0.56–1.75 mmol/L; *p* = 0.003), which was consistent with the difference in NHDL between the two groups (*p* = 0.008). However, the median HDL of the PPDM group (0.87 mmol/L; IQR, 0.69–1.13 mmol/L) was lower than that nPPDM group (1.02 mmol/L; IQR, 0.74–1.31 mmol/L; *p* = 0.040). Moreover, the median of FBG in the PPDM group (7.92 mmol/L; IQR, 6.73–9.87 mmol/L) was significantly higher than that in the nPPDM group (6.90 mmol/L; IQR, 5.61–8.02 mmol/L; *p* < 0.001).

### Risk factors for PPDM by univariate and multivariate analysis

Indicators with statistical differences between the two groups were included in univariate and multivariate Cox proportional hazard analysis to assess the risk factors of PPDM. Before that, we described the continuous variables as categorical variables according to the cut-off values. The results of univariate and multivariate analysis are shown in Table 4. In this study, the junction type, junction angle, NHDL, AI, BMI, FBG, hypertension, and IF were independent predictors of PPDM, with *p*-values all less than 0.05. Compared to the P-B type, the pancreaticobiliary junction in the B-P type and V type were more prone to PPDM, with HR values of 2.735 (95% CI, 1.196–6.255) and 2.494 (95% CI, 1.121–5.545), respectively. In addition, we found that these patients with larger junction angles, higher NHDL or AI or BMI, or FBG, concomitant hypertension, and multiple episodes of inflammation were more likely to develop PPDM.

**Table 4:** Univariate and multivariate Cox proportional hazards analyses

	Univariate analysis		Multivariate analysis	
Variables	HR (95% CI)	*p* value	HR (95% CI)	*p* value
AI, ≥ 1500 vs < 1500	3.623 (2.309–5.682)	**< 0.001**	2.950 (1.698–5.128)	**< 0.001**
BMI, ≥ 25.7vs < 25.7 kg/m^2^	2.878 (1.865–4.439)	**< 0.001**	1.664 (1.019–2.717)	**0.042**
IF, ≥ 2 vs < 2 times	2.899 (1.880–4.484)	**< 0.001**	1.946 (1.130–3.356)	**0.016**
Necrosis, yes vs no	2.012 (1.203–3.367)	**0.008**		
Main location of the lesion				
Head vs neck	1.095 (0.414–2.894)	0.855		
Body vs neck	0.921 (0.328–2.585)	0.875		
Tail vs neck	2.037 (0.808–5.135)	0.132		
Severity				
SAP vs MAP	2.370 (1.323–4.237)	**0.004**		
SAP vs MSAP	2.049 (1.203–3.484)	**0.008**		
Type of junction				
V-type vs P-B-type	3.400 (1.562–7.400)	**0.002**	2.494 (1.121–5.545)	**0.025**
B-P type vs P-B type	7.594 (3.576–16.128)	**< 0.001**	2.735 (1.196–6.255)	**0.017**
Angle of junction, ≥ 42.80 vs < 42.80°	3.690 (2.398–5.682)	**< 0.001**	1.802 (1.024–3.175)	**0.041**
Local complications, yes vs no	1.751 (1.144–2.681)	**0.010**		
Complications type				
APFC vs WON	0.564 (0.170–1.864)	0.347		
PPC vs WON	0.830 (0.136–5.072)	0.841		
ANC vs WON	1.506 (0.349–6.502)	0.583		
TG, ≥ 8.10 vs < 8.10 mmol/L	2.865 (1.862–4.405)	**< 0.001**		
HDL, ≥ 1.10 vs < 1.10 mmol/L	0.565 (0.352–0.907)	**0.018**		
VLDL, ≥ 3.60 vs < 3.60 mmol/L	2.155 (1.325–3.509)	**0.002**		
NHDL, ≥ 5.00 vs < 5.00 mmol/L	2.703 (1.751–4.149)	**< 0.001**	2.198 (1.086–4.444)	**0.029**
FBG, ≥ 9.00 vs < 9.00 mmol/L	2.899 (1.866–4.505)	**< 0.001**	2.370 (1.370–4.115)	**0.002**
Hypertension, yes vs no	2.740 (1.650–4.545)	**< 0.001**	2.817 (1.590–5.000)	**< 0.001**

Bold values in the univariate analysis are further analyzed in the multivariate analysis, and bold values in the multivariate analysis are statistically significant

### Association of independent predictors with the cumulative rate of PPDM

**Fig. 1:**
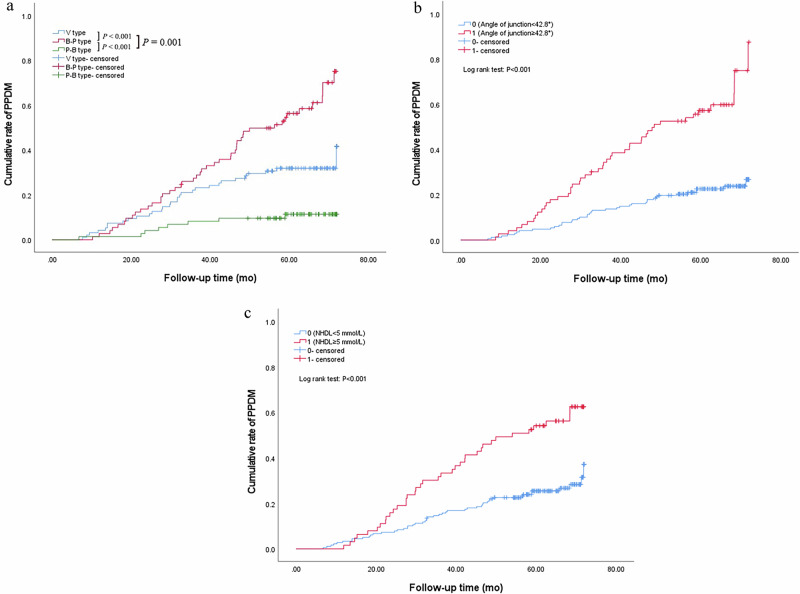
Survival curves by Kaplan-Meier analyses. **a** Comparing the cumulative rate of PPDM among patients with the junction type of B-P, V, and P-B. **b** Comparing the cumulative rate of PPDM between patients with the junction angle ≥ 42.8° and angle < 42.8°. **c** Comparing the cumulative rate of PPDM between patients with NHDL ≥ 5 mmol/L and NHDL < 5 mmol/L. NHDL, non high-density lipoprotein

In this study, in terms of junction type, we found that the cumulative rate of PPDM in patients with B-P type was higher than that of patients with V type (*p* < 0.001), while V type was higher than P-B type (*p* = 0.001, Fig. 1a). The cumulative rate of PPDM in patients with junction angle of 42.8° or greater was higher than that of patients with the angle less than 42.8° (*p* < 0.001, Fig. 1b). For patients with NHDL of 5 mmol/L or greater, the cumulative rate was higher than that of patients with NHDL of less than 5 mmol/L (*p* < 0.001, Fig. 1c). For patients with an AI of 1500 or greater, the cumulative rate was higher than that of patients with an AI of less than 1500 (*p* < 0.001). Patients with a BMI of 25.7 kg/m^2^ or greater had a higher cumulative rate (*p* < 0.001). Similarly, the patients with an FBG of 9.00 mmol/L or greater had a higher cumulative rate (*p* < 0.001). The patients with concomitant hypertension or multiple episodes of inflammation had a higher cumulative rate (*p* < 0.001).

### Development of individualized prediction models based on independent predictors

**Fig. 2:**
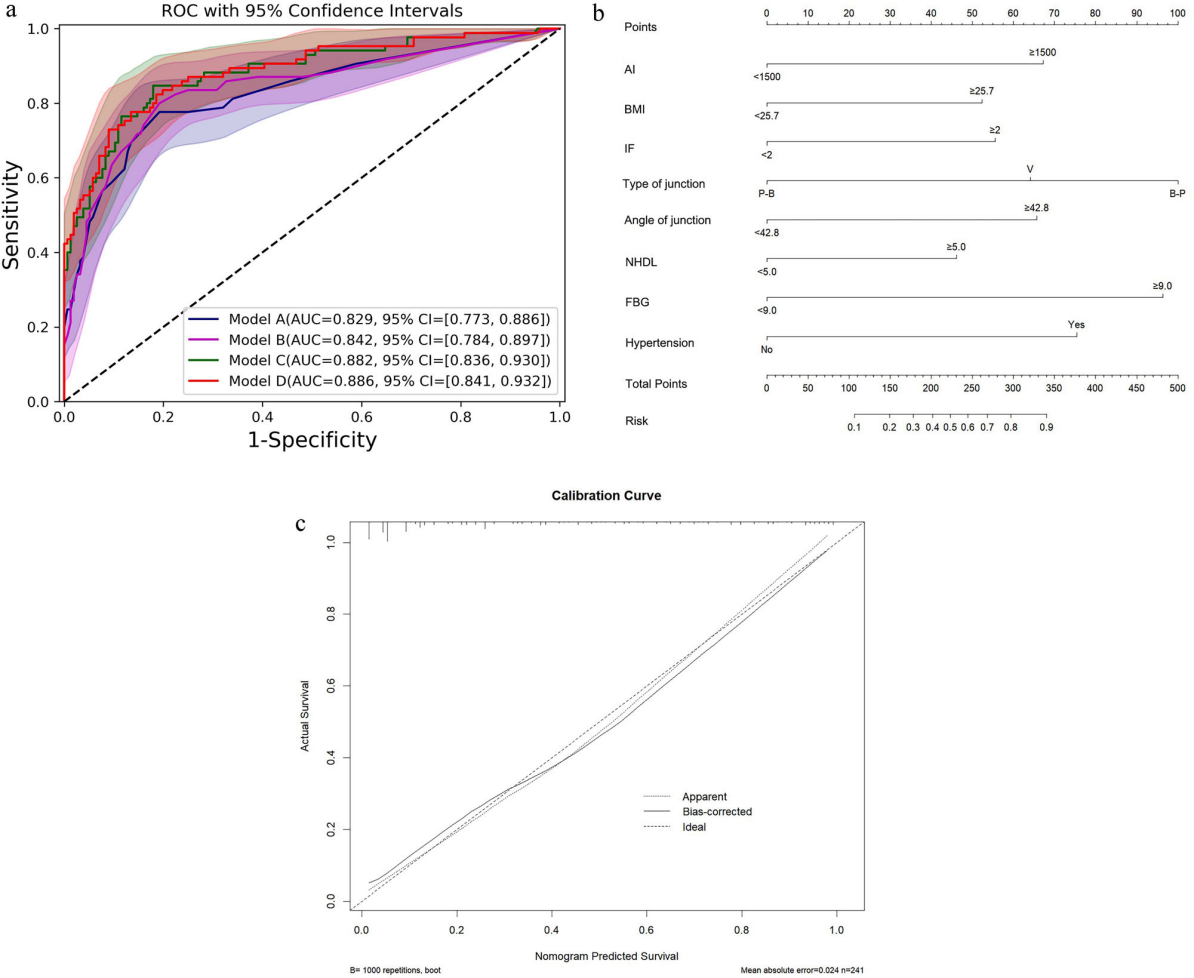
The quantitative predictive models for PPDM. **a** The ROC with 95% CI of each model. **b** The nomogram for the combined model visualization. **c** The calibration curve of the nomogram. AI, alcohol index; BMI, body mass index; IF, inflammation frequency; FBG, fasting blood glucose; ROC, receiver operator characteristic curve; CI, confidence interval

**Fig. 3:**
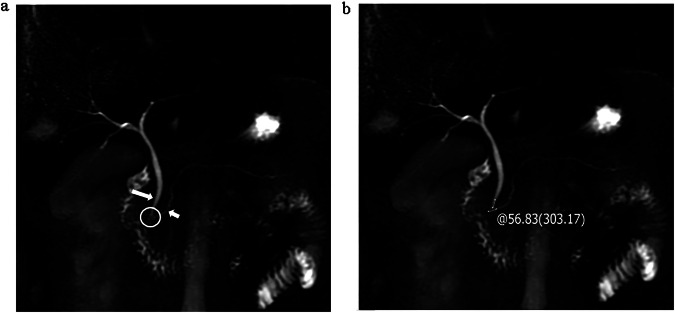
One patient with PPDM. The type of junction (**a**, circle) between the main pancreatic duct (**a**, short arrow) and the common bile duct (**a**, long arrow) is B-P type (100 points) and the angle is 56.83° (66.25 points) (**b**), with AI 1500 (67.5 points), BMI 24.34 (0 points), IF 2 (56.25 points), NHDL 3.41 (0 points), FBG 4.92 (0 points), and no concomitant hypertension (0 points). The total score is about 290 points, and the probability of PPDM occurrence is about 78%

**Fig. 4:**
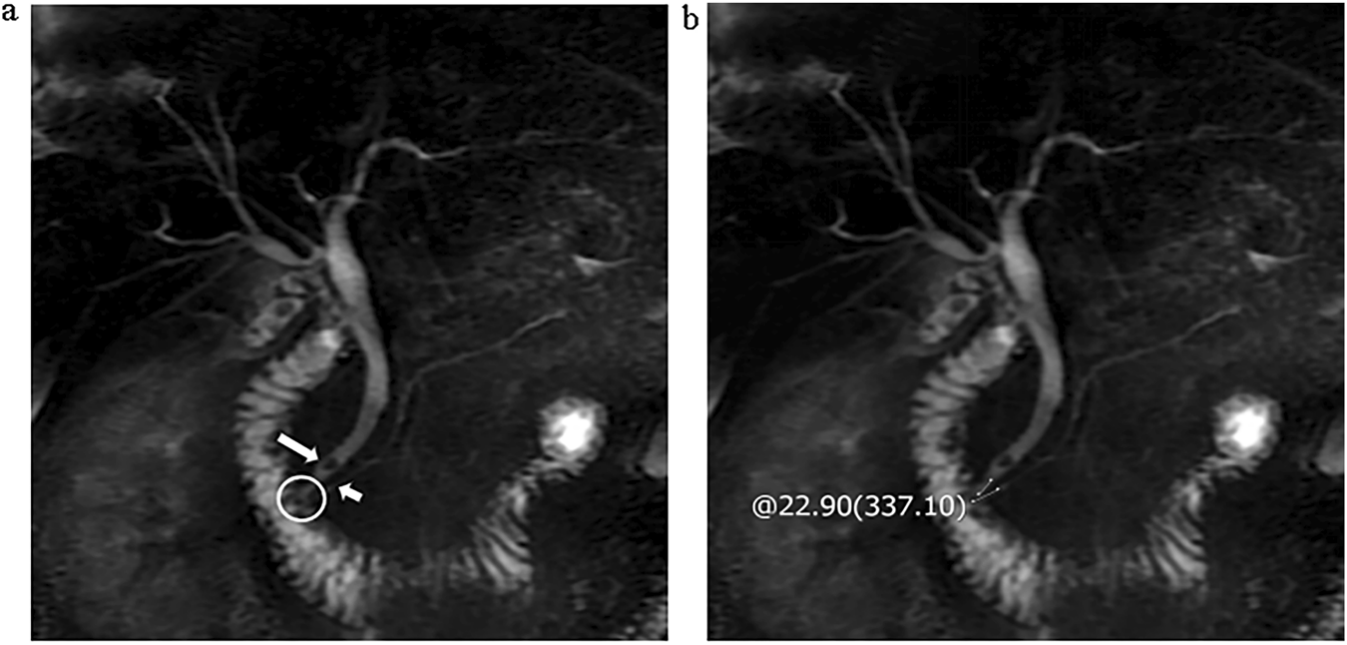
One patient with PPDM. The type of junction (**a**, circle) between the main pancreatic duct (**a**, short arrow) and the common bile duct (**a**, long arrow) is V-shaped (64 points) and the angle is 22.9° (0 points) (**b**), with AI 0 (0 points), BMI 26.37 (52.5 points), IF 2 (56.25 points), NHDL 4.33 (0 points), FBG 9.82 (96.25 points), and concomitant hypertension (75 points). The total score is about 344 points, and the probability of PPDM occurrence is about 90%

**Fig. 5:**
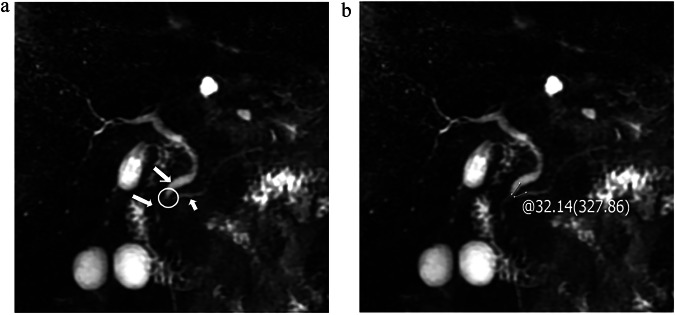
One patient without PPDM. The type of junction (**a**, circle) between the main pancreatic duct (**a**, short arrow) and the common bile duct (**a**, long arrow) is P-B type (0 points) and the angle is 32.14° (0 points) (**b**), with AI 2000 (67.5 points), BMI 21.57 (0 points), IF 1 (0 points), NHDL 2.7 (0 points), FBG 6.50 (0 points), and concomitant hypertension (75 points). The total score is about 142.5 points, and the probability of PPDM occurrence is about 17.5%

Model A was the basic model, which included AI, BMI, IF, FBG, and hypertension, with an AUC of 0.829 (95% CI, 0.773–0.886). Model B integrated lipid metabolism indicator based on Model A, with an AUC of 0.842(95% CI, 0.784–0.897). Model C integrated the features of the pancreaticobiliary junction based on Model A, with an AUC of 0.882 (95% CI, 0.836–0.930). Model D was the combined model of all indicators, with an AUC of 0.886 (95% CI, 0.841–0.932) (Table 5 and Fig. 2a). The further analysis revealed that the angle in B-P type patients has a higher predictive value. For clinically convenient use, model D (the combined model) was visualized as a nomogram (Fig. 2b, c). The calibration curves of the nomogram showed good agreement between the predicted values and the actual values. Based on the nomogram, we predicted the risk of PPDM occurring in AP patients in this study, and the predicted probability was consistent with the actual occurrence of PPDM (Figs. 3–5).

**Table 5:** Comparison of performance efficiency between prediction models

	Model A	Model B	Model C	Model D
AUC	0.829	0.842	0.882	0.886
AUC 95%	0.773–0.886	0.784–0.897	0.836–0.930	0.841–0.932
*p* value^a^	0.191	–	–	–
*p* value^b^	0.002	0.023	–	–
*p* value^c^	0.001	0.005	0.340	–

*AUC* area under the curve

^a^ *p* value represented a comparison with model B

^b^ *p* value represented a comparison with model C

^c^ *p* value represented a comparison with model D

## Discussion

This retrospective study indicates that the long-term complication of PPDM is a concern for patients with a history of AP. However, the reported incidence rates were various in different studies [18, 19]. In a recent systematic review and meta-analysis, the reported incidence of prediabetes or diabetes after AP was about 23% [18]. Bharmal et al [19] found that the incidence rate of new diabetes after AP increased with time. The cumulative incidence was 3.3% at 6 months, 7.2% at 12 months, 9.2% at 18 months, and 11.2% at 24 months of follow-up. Guo et al [20] found that the percentage of PPDM was 31.1%. According to our study, 85 out of 241 eligible patients (35.3%) eventually developed PPDM, with cumulative incidence rates of PPDM at 1, 2, 3, 4, 5, and 6 years of follow-up being 2.9%, 9.5%, 19.5%, 27.4%, 32.4%, and 35.3%, respectively. These results are similar to the previously reported incidence of PPDM.

To the best of our knowledge, this is the first study to explore the value of lipid metabolism and morphological characteristics of the pancreaticobiliary junction in the early prediction of PPDM. The features of the pancreaticobiliary junction could improve the predictive performance of the model and have better predictive value than lipid metabolism indicators. The AUC of the quantitative prediction model was 0.886, and the calibration curve showed that the predicted results were consistent with the actual situation. The probability of PPDM occurrence can be objectively calculated through the nomogram [21], which helps clinical physicians evaluate the risk of patients early, develop personalized treatment plans, and improve the long-term prognosis of patients.

According to our results, the pancreaticobiliary junction in the B-P type was more prone to PPDM. The possible mechanism is that for the B-P type, the common bile duct enters the main pancreatic duct outside the duodenal wall, and the duodenal papillary sphincter cannot act at the confluence site, causing premature convergence of bile and pancreatic juice. The pressure of the bile duct is greater than that of the pancreatic duct, and compared to the P-B type, the main pancreatic duct in the B-P type is closer to the confluence site. Therefore, bile reflux and pancreatic juice extravasation cause more severe mechanical damage to pancreatic tissue, and the risk of pancreatic endocrine dysfunction is greater after the occurrence of AP. In addition, we found that the junction angle was an independent risk factor for PPDM. With the increase of the confluence angle, the incidence rate of PPDM was relatively higher. It is speculated that the damage to pancreatic tissue cells may be caused by the following two factors, leading to insufficient pancreatic endocrine function. On the one hand, an increase in the angle between the confluence of pancreatic and bile ducts can slow down the flow of pancreatic juice into the duodenum or even hinder drainage, thereby increasing the pressure or obstruction of the pancreatic duct. On the other hand, excessive junction angle may weaken the function of the pancreatic sphincter, leading to reflux of bile and pancreatic juice.

Metabolic syndrome, combining obesity, hypertension, hyperglycemia, and hyperlipidemia, is considered one of the risk factors for AP. A previous study has reported that patients with this condition have a higher risk of developing PPDM in the later stage [6], and our study has also obtained similar results. Some studies proposed that NHDL was simpler and more convenient than LDL, and even the strongest blood lipid index to predict coronary heart disease risk [22, 23]. Therefore, our study included NHDL in the analysis and found that NHDL was an independent risk factor for the PPDM. The possible mechanisms are as follows: excessive cholesterol can cause toxicity to pancreatic B cells, affecting normal insulin secretion and leading to elevated blood sugar levels; patients with high cholesterol typically have chronic metabolic disorders, which can lead to excessive lipid accumulation and insulin resistance [24], resulting in elevated blood sugar levels. Our research findings once more confirmed that BMI, FBG, and hypertension were independent risk factors for PPDM. BMI may lead to PPDM through the following pathways. On one hand, higher BMI promotes the accumulation of inflammatory factors, leading to pancreatic microcirculation disorders and endogenous insulin deficiency. On the other hand, the damage to the pancreas and islets β-Cell in AP patients with higher BMI is often more severe, thereby reducing the secretion of endogenous insulin. In addition, excessive body fat deposition in the body is associated with insulin resistance [19, 20, 25]. One of the mechanisms by which patients with high FBG develop PPDM may be related to sustained oxidative stress caused by intermittent hyperglycemia. Persistent oxidative stress continuously stimulates the excessive generation of reactive oxygen species in the body, leading to apoptosis of pancreatic islet cells, increasing the occurrence of individual insulin resistance, and thus increasing the risk of developing PPDM in the later stage [19, 26]. Meanwhile, according to our results, recurrent AP and alcohol abuse can serve as independent predictive indicators for PPDM. Repeated episodes of AP can increase the risk of chronic pancreatitis. Fibrosis of pancreatic tissue leads to a decrease in pancreatic functional cells, resulting in reduced insulin secretion and an increased risk of PPDM [27]. Long-term heavy drinking may lead to impaired pancreatic cell function, insufficient insulin secretion, and, gradually lead to elevated blood sugar levels.

Although our research has made substantial progress, there are still some limitations that need to be addressed in future research. First, although we excluded patients whose fasting or random blood glucose did not fall to normal during hospitalization, we did not measure glycosylated hemoglobin in the first three months after AP, which may have a slight impact on the actual incidence rate of PPDM. Secondly, due to the retrospective study and the unavailability of data, factors such as the family history of diabetes, the dynamic changes of lipid metabolism, and FBG during hospitalization were not included in the analysis, which may affect the comprehensiveness of the results to a certain extent. Therefore, our future research will incorporate more potential influencing factors for comprehensive analysis as far as possible and further increase the sample size to verify the predictive value of junction angle in different junction types.

In summary, the lipid metabolism and morphological characteristics of the pancreaticobiliary junction are additional risk factors for PPDM, and the features of the pancreaticobiliary junction have better predictive value than lipid metabolism indicators. The quantitative model shows moderate predictive performance, which could provide guidance for non-invasive prediction of PPDM and personalized early prevention and treatment strategies for patients.

## Supplementary information

ELECTRONIC SUPPLEMENTARY MATERIAL

## Data Availability

The datasets generated and analyzed during the current study are not publicly available due to our need to expand the sample size and further research based on these datasets, but are available from the corresponding author on reasonable request.

## Abbreviations

AI: Alcohol index
AP: Acute pancreatitis
BMI: Body mass index
CRP: C-reactive protein
FBG: Fasting blood glucose
HDL: High density lipoprotein
IF: Inflammation frequency
IQR: Interquartile range
LDL: Low-density lipoprotein
MRCP: Magnetic resonance cholangiopancreatography
NHDL: Non high-density lipoprotein
PPDM: Post-acute pancreatitis diabetes mellitus
TC: Total cholesterol
TG: Triglycerides
VLDL: Very low-density lipoprotein
