# Supplementary material for: Individualized prediction of post-acute pancreatitis diabetes mellitus by combining lipid metabolism and anatomical features
Source: Insights Imaging. 2025 Jul 31;16:161. doi: 10.1186/s13244-025-02039-w (PMC12314159; doi:10.1186/s13244-025-02039-w)
Supplement: Supplementary file 1 — ELECTRONIC SUPPLEMENTARY MATERIAL [file 13244_2025_2039_MOESM1_ESM.pdf]

Individualized prediction of post-acute pancreatitis diabetes mellitus by combining lipid metabolism and anatomical features

ELECTRONIC SUPPLEMENTARY MATERIAL

Supplementary S1：MR scan equipment and scan parameters

All patients underwent an MR scan with a GE Discovery MR750 3.0 T, 32 channel body phased-array coil. The MRCP adopted a two-dimensional single shot fast spin-echo (SSFSE) by breath-hold, and the parameters were as follows: repetition time (TR) 6000 ms, echo time (TE) 900 ms, slice thickness 40 mm with no gap, FOV 38cm × 38cm, matrix 384×256 and scan time 3 seconds for one slice. The thick-slab sequences were acquired with a radial loop being centred at the level of the pancreaticobiliary junction. Other sequences, such as the axial SSFSE T2-weighted imaging(TR 6000 ms, TE 120 ms, slice thickness 6 mm, gap 1 mm, matrix 320×256, FOV 34cm × 34cm), axial fast recovery fast spin-echo (FRFSE) T2-weighted MR imaging with fat suppression(TR 2609 ms, TE 93 ms, slice thickness 6 mm, gap 1 mm, matrix 384×384, FOV 34cm × 34cm), axial 3D liver acquisition with volume acceleration-fexible (LAVA-Flex) T1-weighted imaging(TR 3.8 ms, TE 1.7 ms, slice thickness 5.2 mm with no gap, matrix 224 × 192, FOV 36cm × 36cm), and axial contrast-enhanced LAVA-Flex T1-weighted imaging(TR 3.8 ms, TE 1.7 ms, slice thickness 5.2 mm with no gap, matrix 224 × 192, FOV 36cm × 36cm) were also performed as routine work.
